# Nutrition outcomes of HIV-infected malnourished adults treated with ready-to-use therapeutic food in sub-Saharan Africa: a longitudinal study

**DOI:** 10.1186/1758-2652-14-2

**Published:** 2011-01-10

**Authors:** Laurence Ahoua, Chantal Umutoni, Helena Huerga, Andrea Minetti, Elisabeth Szumilin, Suna Balkan, David M Olson, Sarala Nicholas, Mar Pujades-Rodríguez

**Affiliations:** 1 Epicentre, Médecins Sans Frontières, 53-55 Rue Crozatier, 75012 Paris, France; 2 Médecins Sans Frontières, Kansanga, Church Zone, Spear Road Plot 2329, Block 244, Kampala, Uganda; 3 Médecins Sans Frontières, 2nd Floor, ABC Place, Wayaki Way, PO Box 39719, Nairobi, Kenya; 4 Médecins Sans Frontières, 8 rue Saint Sabin, 75011 Paris, France

## Abstract

**Background:**

Among people living with HIV/AIDS, nutritional support is increasingly recognized as a critical part of the essential package of care, especially for patients in sub-Saharan Africa. The objectives of the study were to evaluate the outcomes of HIV-positive malnourished adults treated with ready-to-use therapeutic food and to identify factors associated with nutrition programme failure.

**Methods:**

We present results from a retrospective cohort analysis of patients aged 15 years or older with a body mass index of less than 17 kg/m^2 ^enrolled in three HIV/AIDS care programmes in Africa between March 2006 and August 2008. Factors associated with nutrition programme failure (patients discharged uncured after six or more months of nutritional care, defaulting from nutritional care, remaining in nutritional care for six or more months, or dead) were investigated using multiple logistic regression.

**Results:**

Overall, 1340 of 8685 (15.4%) HIV-positive adults were enrolled in the nutrition programme. At admission, median body mass index was 15.8 kg/m^2 ^(IQR 14.9-16.4) and 12% received combination antiretroviral therapy (ART). After a median of four months of follow up (IQR 2.2-6.1), 524 of 1106 (47.4%) patients were considered cured. An overall total of 531 of 1106 (48.0%) patients failed nutrition therapy, 132 (11.9%) of whom died and 250 (22.6%) defaulted from care. Men (OR = 1.5, 95% CI 1.2-2.0), patients with severe malnutrition at nutrition programme enrolment (OR = 2.2, 95% CI 1.7-2.8), and those never started on ART (OR = 4.5, 95% CI 2.7-7.7 for those eligible; OR = 1.6, 95% CI 1.0-2.5 for those ineligible for ART at enrolment) were at increased risk of nutrition programme failure. Diagnosed tuberculosis at nutrition programme admission or during follow up, and presence of diarrhoeal disease or extensive candidiasis at admission, were unrelated to nutrition programme failure.

**Conclusions:**

Concomitant administration of ART and ready-to-use therapeutic food increases the chances of nutritional recovery in these high-risk patients. While adequate nutrition is necessary to treat malnourished HIV patients, development of improved strategies for the management of severely malnourished patients with HIV/AIDS are urgently needed.

## Background

Sub-Saharan Africa is the hardest hit area by the HIV epidemic; it is home to 67% of the estimated 33 million people living with HIV/AIDS worldwide [1]. The highest HIV infection rates are found in southern and east Africa, where adult HIV prevalence can exceed 25%, and food shortages, along with malnutrition and HIV/AIDS, have led some countries to the edge of crisis. Nutritional support is often identified as one of the most immediate and critical needs for people living with HIV/AIDS [2].

Weight loss is common in HIV/AIDS infection. HIV progressively weakens the immune system and impairs nutritional status through the reduction of intake, absorption and use of nutrients, and increased metabolism needs [2,3]. Malnutrition can in turn exacerbate the effects of HIV by increasing susceptibility to AIDS-related illnesses [4,5]. Recommendations have been made to integrate nutrition into the essential package of care, treatment and support for people living with HIV/AIDS. However, effective interventions to achieve this are still lacking.

Several studies have provided evidence of the effectiveness of ready-to-use therapeutic food (RUTF) for treatment of acute malnutrition in HIV-infected and uninfected children [6,7]. However, few data evaluating the effect of RUTF in HIV-infected, malnourished adults are available [8,9], and to our knowledge, no study has investigated factors related to nutrition programme failure in this patient population.

In mid-2006, Médecins Sans Frontières/Doctors Without Borders (MSF), in collaboration with the ministries of health of Uganda and Kenya, began providing RUTF to all severely malnourished HIV patients followed in the HIV/AIDS programmes of Arua in rural north-western Uganda, Homa Bay in rural north-eastern Kenya, and Mathare slum in Nairobi, Kenya. The RUTF provided is an energy-dense spread of peanut, milk powder, oil and sugar, highly fortified with micronutrients, originally designed for the treatment of childhood severe acute malnutrition. The objectives of this analysis were to evaluate the nutritional outcomes of HIV-infected malnourished adults treated with RUTF in these three MSF-supported HIV/AIDS programmes in Africa from 2006 to 2008, and to identify factors associated with nutrition treatment failure.

## Methods

### HIV care and treatment programme

The Arua Regional Referral Hospital in Uganda serves as the tertiary health care facility for seven districts covering a rural population of more than 2 million people. Homa Bay District Hospital in Kenya is a referral hospital covering a rural area of around 300,000 inhabitants. In Arua and Homa Bay, MSF, in collaboration with the respective country's Ministry of Health, provides outpatient and inpatient HIV and tuberculosis (TB) care. The Mathare clinic is a stand-alone clinic, located in Nairobi, providing HIV and TB treatment and care for people living in the slum. When necessary, patients are referred for hospitalization.

All diagnosed HIV-infected patients were eligible for enrolment in the Arua and Mathare programmes, but only patients diagnosed with World Health Organization (WHO) stage 3 or 4 conditions were enrolled in Homa Bay. Eligibility criteria for starting combination antiretroviral therapy (ART) were those recommended in the 2006 WHO guidelines for scaling up ART in resource-poor settings: all patients with WHO clinical stage 4, or patients with CD4 counts of less than 200 cells/mm^3^. CD4 cell counts were monitored at ART start, at six months, and yearly after the first year of therapy. No routine viral load monitoring was performed.

When a new patient is eligible for ART, he/she receives three pre-ART counselling sessions: first, on the day of ART eligibility assessment; second, two weeks later; and third, one to two weeks thereafter. The process takes between 15 to 30 days, but may vary according to patient clinical status or readiness to initiate ART.

### Nutrition programme

Malnourished adults (aged 15 years or older in Kenyan and 18 years or older in Ugandan programmes) enrolled in the HIV/AIDS programmes received therapeutic feeding if their body mass index (BMI) was less than 17 kg/m^2 ^or they had bilateral pitting oedema at the lower extremities. Patients received four sachets of RUTF (2000 kcal; Plumpy'nut^®^, Nutriset, Malaunay, France) per day in the outpatient clinic, and were clinically assessed every two weeks or monthly before renewal of the RUTF prescription.

The predefined nutrition programme (NP) exit criterion was BMI ≥18 kg/m^2 ^with no oedema for at least two consecutive weeks (defined as "cured" according to the programme's definition). After enrolment in the NP, patients meeting the predefined NP exit criterion at any time were discharged from the programme after full clinical review. Patients unresponsive to nutritional therapy after six months of treatment (not reaching BMI ≥18 kg/m^2^; i.e., not meeting the predefined NP exit criterion) were reviewed by a physician for further investigations and management. After clinical assessment to exclude presence of undiagnosed pathologies, they were discharged from the NP. They were also referred to patient support groups and given normal food support (corn-soya blend, beans and oil) through aid agencies. This latter group of patients was defined as discharged "uncured" according to the programme's definition.

### Data collection

At each patient's visit, anonymous individual HIV and nutritional data were routinely collected on standardized forms and entered into FUCHIA software database (Epicentre, Paris, France) and EpiData (version 3.1, EpiData Association, Odense, Denmark). Data collected included sex, age, enrolment dates in the HIV and nutrition programmes, follow-up visit dates, ART regimen prescribed during the visit, weight, height, BMI at NP admission and discharge, presence of oedema, opportunistic infections diagnosed at each visit, CD4 count, blood collection dates, and NP outcome categorized as cured, discharged uncured, defaulted, treatment stopped, transferred to another HIV programme, or death.

### Study design and population

We retrospectively analyzed the outcomes of all HIV-positive adults followed in the three HIV/AIDS care programmes who were eligible for nutritional rehabilitation and treated in the NP with RUTF. Pregnant women and HIV-positive patients enrolled in HIV care before the availability of RUTF were excluded from the analysis.

This multicentre study was based on analysis of routinely collected, patient monitoring data from the three programmes. In agreement with the Ministry of Health of each country, clinical, therapeutic and laboratory patient data are routinely collected for patient and programme monitoring; as such, no formal ethics approval from institutional review boards and/or written patient consent were required. Local health authorities were informed of the data analysis and potential publication of findings, with written approval obtained from the Kenyan health authorities and verbal approval from the Ugandan health authorities. Databases were anonymized before data compilation and analysis, and findings were shared with our partners in the health ministries.

### Definitions and data analysis

A patient was considered severely malnourished at admission if BMI was less than 16 kg/m^2 ^and moderately malnourished if BMI was 16-17 kg/m^2^. New patients were those admitted into the NP within one month of enrolment in the HIV/AIDS care programme. Patients were classified according to their ART status at NP admission as: not eligible for ART; on ART; eligible and started on ART at or after NP admission; and eligible but never started on ART. Defaulters from nutrition care were patients who missed two or more consecutive NP visits.

NP outcomes were defined as: programme success (patients discharged from the NP and "cured" according to the predefined NP exit criterion); programme failure (patients discharged "uncured" according to the predefined NP exit criterion, on NP care for six months or more, defaulting from NP, or dead); or other (patients who experienced intolerance to RUTF, stopped nutritional therapy on request or for other reasons, or were transferred to another HIV programme). The overall programme failure rate was calculated by dividing the total number of failure outcomes (discharged uncured, died, defaulted, or still in the NP for six months or more) by the total number of patients admitted into the NP, excluding those who were receiving nutrition therapy for more than 6 months and were still followed in the NP. In sensitivity analyses, patients who stopped nutrition therapy for intolerance or other reasons were also considered NP failures.

We only considered the first recorded episode of malnutrition for each patient. Data were described using standard statistics for continuous and categorical variables, and compared with non-parametric, χ^2^, or Fisher's exact tests, as appropriate.

To investigate associations with NP failure, factors significantly associated with the outcome in univariate analyses (p < 0.20) were included in a multiple logistic regression model [10]. The final model was obtained through the backward-stepwise procedure and the goodness-of-fit χ^2 ^test was used to determine the fit of the model [11]. Patients still on nutritional therapy and in the NP for 6 months or less were excluded from this analysis because they did not yet have an NP outcome.

To investigate whether the results were robust to changes in our definition of failure, we performed two sensitivity analyses using alternative programme failure definitions. First, we excluded patients with NP outcome defined as "other" (Model 1). Second, we classified patients with intolerance to RUTF and those who stopped nutritional therapy for other reasons as "programme failure", and those referred to another programme as "programme success" (Model 2). All analyses were performed using Stata 9.2 (Stata Corp., College Station, TX, USA).

## Results

### Patient characteristics at nutrition programme admission

Overall, 8685 HIV-positive adults were enrolled in the three HIV care programmes between NP start (March 2006 for Kenyan and July 2006 for Ugandan programmes) and August 2008. A total of 1340 of 8685 (15.4%) HIV-positive adults were eligible for RUTF treatment and enrolled in the NP. Of those admitted and enrolled into the NP, 1057 (78.8%) patients had been discharged at the time of the analysis, and the remaining 283 (21.2%) were still receiving NP therapy, 234 of these for less than six months and 49 for six months or more. The 234 patients who had received NP therapy for less than six months and had not been discharged were excluded from further analyses, and the 49 patients who were receiving NP for six months or more were classified as "uncured".

We describe the characteristics at NP admission for the 1106 patients (Table 1). A total of 56.7% (627 of 1106) of patients were women, and median age was 33 years (IQR 28-40). Seventy-seven percent were enrolled in the NP within one month of admission in the HIV/AIDS care programme. Patients already followed in the HIV programme for more than one month were in care for a median of 2.3 months (IQR 1.5-4.7). At admission, median BMI was 15.8 kg/m^2 ^(IQR 14.9-16.4), 617 (55.8%) patients had severe malnutrition (<16 kg/m^2^), and 489 (44.2%) had moderate malnutrition (16-17 kg/m^2^). Median CD4 count at NP admission was 114 cells/mm^3 ^(IQR 37-268) (n = 806), and 65.9% (705 of 1070) of patients were in HIV clinical stage 3 or 4. At enrolment, the most frequently diagnosed opportunistic infections were TB (n = 194), chronic diarrhoea (n = 113), and fever of unknown aetiology (n = 82).

**Table 1:** Characteristics of HIV-infected adults at admission, by outcome at discharge, in three nutritional therapy programmes in Kenya and Uganda, 2006-2008

Characteristics	Cured n = 524 (47.4%)	Not cured n = 149 (13.5%)	Defaulted n = 250 (22.6%)	Died n = 132 (11.9%)	**Other**^**a **^**n = 51 (4.6%)**	Total N = 1106
**Demographic factors**						
Women (%)	323 (61.6)	79 (53.0)	128 (51.2)	64 (48.5)	33 (64.7)	627 (56.7)^**c**^
Median age, years [IQR]	32 [27-40]	35 [29-40]	33 [27-40]	34 [30-42]	35 [27-44]	33 [28-40]^**f**^
**Follow up in HIV care**						
New patients (%)	398 (76.0)	116 (77.9)	208 (83.2)	102 (77.3)	32 (62.8)	856 (77.4)^**d**^
In HIV care (%)	126 (24.0)	33 (22.1)	42 (16.8)	30 (22.7)	19 (37.2)	250 (22.6)
**Nutritional status**^**a**^						
BMI, kg/m^2^, median [IQR]	16.0 [15.4-16.5]	15.5 [14.6-16.3]	15.6 [14.3-16.3]	15.2 [14.0-16.2]	15.5 [14.3-16.4]	15.8 [14.9-16.4]^**e**^
Severe malnutrition, BMI <16 kg/m^2 ^(%)	245 (46.8)	96 (64.4)	157 (62.8)	91 (68.9)	28 (54.9)	617 (55.8)^**e**^
Moderate malnutrition, BMI 16-17 kg/m^2 ^(%)	279 (53.2)	53 (35.6)	93 (37.2)	41 (31.1)	23 (45.1)	489 (44.2)
**Clinical & immunological factors**						
**Non-cumulative HIV clinical stage (%)**	n = 508	n = 143	n = 242	n = 128	n = 49	**n = 1070 **^**c**^
Asymptomatic	40 (7.9)	10 (7.0)	20 (8.3)	12 (9.4)	5 (10.2)	87 (8.1)
1 or 2	149 (29.3)	48 (33.5)	50 (20.7)	21 (16.4)	10 (20.4)	278 (26.0)
3	248 (48.8)	65 (45.5)	119 (49.2)	65 (50.8)	23 (46.9)	520 (48.6)
4	71 (14.0)	20 (14.0)	53 (21.8)	30 (23.4)	11 (22.5)	185 (17.3)
**CD4 cell counts, cells/mm**^**3**^	n = 411	n = 119	n = 155	n = 83	n = 38	**n = 806 **^**e**^
Median [IQR]	122 [46-272]	188 [86-360]	94 [24-232]	39 [17-126]	74 [42-206]	114 [37-268]
<50 (%)	111 (27.0)	25 (21.0)	61 (39.3)	46 (55.5)	14 (36.8)	257 (31.9)
50-200 (%)	161 (39.2)	36 (30.3)	46 (29.7)	24 (28.9)	14 (36.8)	281 (34.9)
>200 (%)	139 (33.8)	58 (48.7)	48 (31.0)	13 (15.7)	10 (26.4)	268 (33.2)
**ART status (%)**						
Not eligible for ART	132 (25.2)	56 (37.6)	89 (35.6)	29 (22.0)	10 (19.6)	316 (28.6)^**e**^
On ART	68 (13.0)	17 (11.4)	23 (9.2)	20 (15.2)	5 (9.8)	133 (12.0)
ART started at/after admission	298 (56.8)	70 (47.0)	46 (18.4)	36 (27.3)	20 (39.2)	470 (42.5)
Eligible but no ART	26 (5.0)	6 (4.0)	92 (36.8)	47 (35.5)	16 (31.4)	187 (16.9)
**ART regimen (%)**^**b**^	n = 68	n = 17	N = 23	n = 20	n = 5	**n = 133 **^**f**^
2 NRTI + 1 NNRTI	64 (94.1)	17 (100)	22 (95.7)	20 (100)	5 (100)	128 (96.2)
Second-line therapy	1 (1.5)	0 (0.0)	1 (4.3)	0 (0.0)	0 (0.0)	2 (1.5)
ART interrupted	3 (4.4)	0 (0.0)	0 (0.0)	0 (0.0)	0 (0.0)	3 (2.3)

^a^No patients had recorded presence of bilateral oedema

^b^Among the 133 patients already receiving ART at NP admission

^c^P < 0.01

^d^P = 0.02

^e^P < 0.0001

^f^P ≥ 0.05

ART - antiretroviral therapy; BMI - body mass index; IQR - interquartile range; NRTI - nucleoside reverse transcriptase inhibitor; NNRTI - non-nucleoside reverse transcriptase inhibitor

A total of 790 of 1106 (71.4%) patients were classified as eligible for ART according to the recorded clinico-immunological information. Of those eligible for treatment, 133 initiated ART before, and 470 at or after, NP admission; 187 never received ART. Most patients on ART prior to NP admission received a combination of two nucleoside reverse transcriptase inhibitor (NRTI) and one non-NRTI (NNRTI) drugs for a median of 0.5 months (IQR 0-2.6).

Median age and sex distribution were independent of ART status (data not presented). However, median BMI at admission was slightly lower in patients who were eligible for but never started ART (15.4 kg/m^2^; IQR 14.2-16.3) than in the other groups: 15.9 kg/m^2 ^(IQR 14.5-16.4) for those who initiated ART before NP entry; 15.8 kg/m^2 ^(IQR 14.8-16.3) for those who initiated ART at or after NP admission; and 16.0 kg/m^2 ^(IQR 15.2-16.6) for those ineligible for ART (P = 0.002).

### Nutritional outcomes

Of the 1106 patients admitted into the NP and discharged, 524 (47.4%) were considered cured according to the predefined NP exit criterion (programme success), 149 (13.5%) discharged uncured, 250 (22.6%) defaulted from NP care, 132 (11.9%) died, 26 (2.4%) transferred to another programme, and 25 (2.3%) stopped RUTF due to treatment intolerance or other reasons (Table 2). The overall programme failure rate was 48.0% (531 of 1106); if patients who transferred to another programme or who stopped NP were also considered, programme failure rate was 52.6% (582 of 1106).

**Table 2:** Characteristics of HIV-infected adults at discharge from three nutritional therapy programmes in Kenya and Uganda, by nutrition outcome, 2006-2008

Patient characteristics	Cured	Not cured	Defaulted	Died	**Other**^**a**^	Total
	**n = 524 (47.4%)**	**n = 149 (13.5%)**	**n = 250 (22.6%)**	**n = 132 (11.9%)**	**n = 51 (4.6%)**	**N = 1106**
**Demographic factors**						
**Women **(%)	323 (61.6)	79 (53.0)	128 (51.2)	64 (48.5)	33 (64.7)	627 (56.7) ^**b**^
**Age**, years, median [IQR]	32 [27-40]	35 [29-42]	33 [27-40]	34 [30-42]	35 [27-44]	33 [28-40] ^**c**^
**NP follow-up time**, months, median [IQR]	3.7 [2.2-6.1]	7.1 [5.9-9.6]	2.3 [1.0-3.9]	1.6 [0.8-2.8]	2.8 [0.9-5.6]	3.3 [1.7-6.2] ^**c**^
**Nutritional indicators**						
**Daily weight gain, g/kg/d**, median [IQR]	1.6 [1.0-2.6]	0.3 [0.1-0.6]	0 [-0.4-0.3]	0 [-1.1-0]	0.05 [-0.5-1]	0.8 [0-1.8]^**c**^
**Weight gain**, kg median [IQR]	8 [5.5-11]	3 [1-5]	0 [-2-1]	0 [-3-0]	1 [-2.5-4]	4 [0-8] ^**c**^
**BMI**, kg/m^2^	n = 524	n = 102	n = 230	n = 126	n = 47	**n = 1029**
Median [IQR]	18.7 [18.2-19.5]	16.7 [15.8-17.3]	15.2 [14.0-16.2]	14.9 [13.4-16.0]	15.8 [14.5-17.2]	17.7 [15.6-18.8] ^**c**^
**Clinical & immunological factors**						
**Non-cumulative HIV clinical stage (%)**	n = 490	n = 89	n = 142	n = 21	n = 42	**n = 784 **^**c**^
Asymptomatic	47 (9.6)	7 (7.9)	13 (9.2)	1 (4.8)	6 (14.3)	74 (9.4)
1 or 2	280 (57.1)	60 (67.4)	33 (23.2)	5 (23.8)	13 (31.0)	391 (49.9)
3	129 (26.4)	21 (23.6)	68 (47.9)	8 (38.1)	15 (35.7)	241 (30.7)
4	34 (6.9)	1 (1.1)	28 (19.7)	7 (33.3)	8 (19.0)	78 (10.0)
**CD4 cell count**, cells/mm^3^	n = 173	n = 47	n = 63	n = 44	n = 18	**n = 345**
Median [IQR]	218 [106-363]	292 [201-454]	96 [33-214]	36 [16-129]	91.5 [45-431]	188 [58-349] ^**c**^
<200 (%)	78 (45.1)	11 (23.4)	44 (69.8)	36 (81.8)	10 (55.6)	179 (51.8) ^**c**^

^a^Other group includes patients who stopped RUTF for intolerance or other reason and those referred to another programme

^b^P < 0.01

^c^P < 0.0001

BMI - body mass index; IQR - interquartile range; NP - nutrition programme

Cured patients were discharged from the NP after a median of 3.7 months (IQR 2.2-6.1) of treatment (Table 2). At discharge, their daily weight gain since NP admission was 1.6 g/kg/day (IQR 1.0-2.6), median weight gain achieved since NP admission was 8 kg (IQR 5.5-11.0), and 57.1% (280/490) were in HIV clinical stage 1 or 2. Patients uncured after nutritional therapy had been treated for a median of 7.1 months (IQR 5.9-9.6). A total of 67.4% (60 of 89) of these patients were in HIV clinical stage 1 or 2, with median CD4 count of 292 cells/mm^3 ^(IQR 201-454), and no CD4 cell gain was observed during NP follow up.

Median BMI at discharge was 16.7 kg/m^2 ^(IQR 15.8-17.3) and daily weight gain since NP admission was 0.3 g/kg/day (IQR 0.1-0.6). Patients who defaulted from NP care or died had received nutritional therapy for less than three months and were severely malnourished (median BMI at last visit 15.2 kg/m^2 ^[IQR 14.0-16.2] and 14.9 kg/m^2 ^[IQR 13.4-16.0], respectively). Furthermore, 67.6% (96 of 142) of defaulting patients and 71.4% (15 of 21) of deaths were in HIV clinical stage 3 or 4, and were severely immunosuppressed at last visit (median CD4 counts 96 cells/mm^3 ^[IQR 33-214] and 36 cells/mm^3 ^[IQR 16-129], respectively).

**Figure 1:**
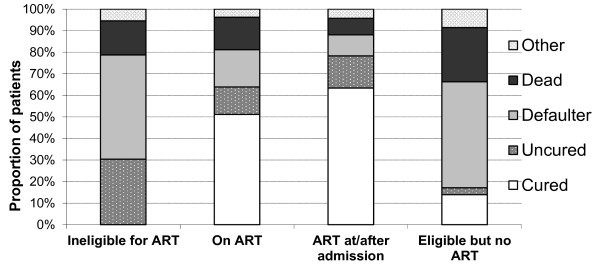
Outcomes of HIV-infected patients treated in three nutritional therapy programmes in Kenya and Uganda, by antiretroviral therapy status*, 2006-2008. *N = 1106; proportion of patients by nutritional therapy outcome is presented for each category of ART status at admission in the nutrition programme. ART - antiretroviral therapy

When comparing ART eligibility among the patients discharged, those eligible for but never started on ART had the lowest median BMI (15.4 kg/m^2^; IQR 14.0-16-6) at discharge with no overall weight gain or daily weight gain compared with other groups. These patients had the highest death and default rates and lowest cure rates (25.1%, 49.2%, and 13.9%, respectively) (Figure 1). After a median length of stay in the NP of 1.9 months (IQR 0.8-2.9), median CD4 count at last visit was 70 cells/mm^3 ^(IQR 24-200), and 77% were in clinical stage 3 or 4. In contrast, patients who were eligible for and initiated ART during NP care had the highest cure rate (63.4%), weight gain (6.5 kg; IQR 3.0-10.0), and BMI (18.3 kg/m^2^; IQR 16.8-19.1) at discharge.

### Factors associated with nutrition programme failure

Risk factor analysis was based on available information from 507 adults successfully treated and 509 patients who failed nutritional therapy. We excluded 234 patients who received nutritional therapy for less than six months.

Men (adjusted OR [OR_a_] 1.5, 95% CI 1.2-2.0) and patients severely malnourished at NP admission (OR_a _2.2, 95% CI 1.7-2.8) were at increased risk of NP failure (Table 3). Furthermore, compared with patients who were already receiving ART at NP admission, patients who never initiated therapy despite being eligible (OR_a _4.5, 95% CI 2.7-7.7) and patients not eligible for ART at NP admission (OR_a _1.6, 95% CI 1.0-2.5) were both at increased risk of NP failure. Patients eligible for and started on ART at or after NP admission were less likely to fail nutritional therapy (OR_a _0.6, 95% CI 0.4-0.9).

**Table 3:** Factors associated with nutrition programme failure among HIV-infected adults treated in Kenya and Uganda, 2006-2008

Factors	Adjusted (Model 1)		Adjusted (Model 2)	
	**OR (95% CI)**	**P value**	**OR (95% CI)**	**P value**
**Treatment cohort**				
Arua	**1.0**	**0.01**	**1.0**	**0.007**
Homa Bay	**1.5 (1.0-2.3)**		**1.7 (1.1-2.4)**	
Mathare	**0.7 (0.4-1.0)**		**0.8 (0.5-1.1)**	
**Period of admission in the NP**				
Years 2005 - 2006	1.0	0.1	1.0	0.1
January-June 2007	0.8 (0.5-1.2)		0.8 (0.6-1.2)	
July 2007-June 2008	1.1 (0.8-1.6)		1.1 (0.8-1.2)	
**In the HIV programme**				
Patients already in care	1.0	0.5	1.0	0.5
New patients	1.1 (0.8-1.6)		1.1 (0.8-1.6)	
**Age at NP admission**, years				
≥45	1.0	0.2	1.0	0.4
30-44	1.0 (0.6-1.5)		1.0 (0.6-1.4)	
15-29	0.7 (0.4-1.2)		0.8 (0.5-1.2)	
**Gender**				
Women	**1.0**	**0.001**	**1.0**	**0.001**
Men	**1.6 (1.2-2.1)**		**1.5 (1.2-2.0)**	
**Malnutrition at admission**				
Moderate	**1.0**	**<0.0001**	**1.0**	**<0.0001**
Severe	**2.2 (1.7-3.0)**		**2.2 (1.7-2.8)**	
**Recorded clinical diagnoses**				
TB at NP admission	0.9 (0.6-1.3)	0.7	0.9 (0.6-1.3)	0.6
TB diagnosed during NP follow up	1.0 (0.7-1.4)	0.9	1.0 (0.7-1.4)	0.9
Extensive candidiasis at NP admission	1.4 (0.6-3.4)	0.4	1.2 (0.5-2.6)	0.7
Diarrhoea at NP admission	1.3 (0.8-2.0)	0.3	1.3 (0.8-2.0)	0.3
**ART status at admission**				
On ART	**1.0**	**<0.0001**	**1.0**	**<0.0001**
Eligible but never started on ART	**6.2 (3.5-11.1)**		**4.5 (2.7-7.7)**	
Eligible & ART initiated at or after NP admission	**0.5 (0.3-0.8)**		**0.6 (0.4-0.9)**	
Ineligible for ART	**1.4 (0.9-2.3)**		**1.6 (1.0-2.5)**	

Model 1: Results from analysis where deaths, lost to follow up and uncured were classified as NP failure; and patients with NP outcome defined as "other" were excluded from the model.

Model 2: Results from analysis where deaths, lost to follow up, uncured, patients with intolerance to RUTF and those who stopped nutritional therapy for other reasons were classified as "programme failure".

ART - antiretroviral therapy; NP - nutrition programme; OR - odds ratio; TB - tuberculosis

Diagnosed TB at NP admission or during follow up, and presence of diarrhoeal disease or extensive candidiasis at admission, were unrelated to the risk of NP failure. P value from the goodness-of-fit test for the final regression model was 0.11. The observed results were robust to the sensitivity analyses using the alternative definitions of NP failure and success (data not shown).

## Discussion

In this evaluation of nutritional outcomes of HIV-infected malnourished adults treated with RUTF in three sub-Saharan African HIV/AIDS programmes, 15% of all patients enrolled for HIV care were diagnosed with acute malnutrition and received therapeutic nutritional rehabilitation.

One in two patients was severely malnourished at NP admission, and approximately three in four were admitted into the NP within one month of HIV programme enrolment. At NP admission, 64% of patients had advanced HIV clinical disease and were severely immunosuppressed (<200 cells/mm^3^). Furthermore, severely malnourished patients had a two-fold increased risk of NP failure compared with moderately malnourished patients, stressing the importance of closely monitoring the nutritional status of HIV patients, treating malnutrition at early stages, and increasing early access to HIV/AIDS care.

An important finding of this evaluation was that 70% of patients were eligible for ART at NP admission, but one in five were never initiated on therapy, probably due to several reasons, such as delay of ART initiation for TB co-infected patients after completion of the TB intensive-phase treatment, delayed blood test results or patient refusal. As expected, many of the patients who needed but never received ART died (26%) or defaulted (50%) from care shortly after NP enrolment and had a 4.5-fold increased risk of nutritional therapy failure, including death, compared with patients already on ART. These findings highlight the importance of integrating HIV and nutrition care to carefully monitor patient eligibility for ART and initiate therapy early to prevent deaths.

This study also showed that that the risk of NP failure was 1.6 times higher in patients not eligible for ART at NP enrolment than in those already on ART at admission. This finding could reflect the existence of undiagnosed severe clinical conditions and/or severe HIV disease, and suggests that ART should be provided to all malnourished HIV-infected patients regardless of their theoretical eligibility status to ART.

Furthermore, we observed that patients who initiated ART while receiving nutrition treatment had lower risk of NP failure than those already on ART. This is supported by evidence from a prospective study assessing acceptability and effectiveness of a locally produced RUTF in HIV-infected, chronically ill adults in Malawi [8], where patients commencing ART prior to or while on nutritional therapy experienced greater weight and BMI gains. Furthermore, the greater frequency of visits and support of counsellors at the time of ART start could help reinforce adherence, not only to ART but also to nutritional therapy [12]. This could therefore partly explain the better nutritional outcomes observed in the group of patients who started ART while receiving nutrition support.

Overall, 50% of patients were cured after receiving nutritional treatment for a median of four months and achieved an average weight gain of 1.6 g/kg/day. HIV-positive adults have higher energy requirements than healthy non-HIV-infected individuals [2,13,14] due to increased resting energy expenditure, presence of fever and infection, diarrhoea and vomiting, and the need for growth and weight recovery.

The RUTF in this study was originally developed to treat severe acute malnutrition in HIV-uninfected children [15,16]. Studies in Malawi have reported cure rates of 86% and 75% for HIV-negative and HIV-positive children receiving the same RUTF, respectively [6,17]. Since only a paediatric formulation of this RUTF is currently available, it is also used to treat malnourished HIV-positive adults, but it might not be the best nutritional option for this patient population. Previous studies in HIV-positive adults showed that the quantity of RUTF intake is positively associated with weight and BMI recovery [8]; therefore, poor adherence in some of the patients could partly explain the low cure rates observed.

A recent qualitative study of RUTF acceptability among HIV-positive adults in Homa Bay, Kenya, showed that only half of the patients receiving the product actually complied with the full prescribed dose (2000 kcal/day), due to poor taste, diet boredom, bulky weight (~12 kg; two-week supply needed to be carried by the patient, and patients would tend to reduce their daily intakes to ensure that the amount received lasted until the next scheduled clinic visit), and sharing of supply with other household members [18]. Further research is needed to design and evaluate a RUTF better adapted to the specific needs of HIV-positive adults that might help improve their nutritional status.

The highest cure rate was observed for patients receiving RUTF and who were eligible for and initiated ART at or after NP admission. In Malawi, a randomized controlled trial compared outcomes of food supplementation in HIV-infected adults initiating ART and receiving either RUTF (260 g/day, 1360 kcal/day) or corn-soya blend (374 g/day, 1360 kcal/day) [9]. Patients in the RUTF group achieved mean overall weight gain of 5.6 kg, with median BMI of 19.0 kg/m^2 ^after 3.5 months of treatment. However, the proportion of patients with moderate malnutrition was higher (67%), and all patients were treated with ART, in contrast to our study patients. In addition, no significant difference in mortality was observed between the two groups. Further studies are needed to evaluate the true impact on mortality of nutritional rehabilitation among patients initiating ART [19].

More than one in three patients died or defaulted from care during the first three months of treatment, and it is likely that many of the defaulting patients died shortly after treatment initiation. Similarly, a previous study reported an overall 27% death-defaulter rate in Malawi [8], confirming that severe weight loss is associated with both occurrence of severe opportunistic infections and death [4,20-23].

In our study, men had an odds of failure 1.5 times higher than women. Knowing that in our programmes, men tend to access HIV care at a more clinically and/or immunologically advanced stage of disease than women [24], a higher risk of nutritional failure or death therefore exists in men. Gender differences in patient compliance to nutritional treatment and/or ART could also explain our findings.

The higher risk of NP failure observed in patients treated in the Homa Bay programme could be explained by their more advanced stage of HIV disease at enrolment. Indeed, at the time of the study, only advanced WHO stage 3 and 4 patients were enrolled in the Homa Bay HIV cohort and entered into the database. For patients with less advanced HIV infection, clinical information was not monitored with a computerized system. Therefore, these patients have not been included in this analysis.

This retrospective cohort study was based on the analysis of routinely collected data from three HIV care programmes. Indeed, certain types of information, such as CD4 cell counts at NP admission, were missing for some of the patients. Nevertheless, efforts were made in the programmes to ensure and maintain the quality and completeness of the data collected. Checks at data entry and regular verifications of inconsistencies were routinely performed.

Data from three different programmes were analyzed. However, all used the same criteria for inclusion to and discharge from the NP, applied the same criteria for ART initiation, and provided the same antiretroviral regimens. Information on household food availability, dietary intake from other sources, or patient compliance to nutrition therapy and/or ART was not available and could have biased the results of our risk-factor analysis. In addition, the absence of a comparison group did not allow investigating the additional benefit of providing RUTF to patients also receiving ART.

## Conclusions

We have reported here on our first experience in treating severely malnourished HIV-infected adults with RUTF in three routine, home-based therapeutic feeding programmes in sub-Saharan Africa. In these programmes, 15% of the HIV patients in care required nutritional rehabilitation, and cure rates varied widely from 14% to 67%, according to the patient ART status at NP admission.

Despite the limitations of this observational study, our findings suggest that the administration of nutrition therapy, in conjunction with an early start of ART, might increase the chances of nutritional recovery in severely malnourished HIV patients. Furthermore, this study shows that nutritional support with RUTF may be more effective when provided to patients at earlier stages of malnutrition. While adequate nutrition is necessary to treat malnourished HIV patients and maximize the benefit of ART, there is still a need to clearly define and evaluate the most effective ways of administering such care.

## Competing interests

The authors declare that they have no competing interests.

## Authors' contributions

LA and MPR designed the study, analyzed and managed data, interpreted results, and wrote the manuscript. CU and HH assisted with the study in the field, and contributed to the interpretations of results. AM, ES, SB and DMO contributed to the design of the study, interpretation of results, and critical revision of the manuscript. SN contributed to data management and analyses. All authors read and approved the final manuscript.
